# Land Surface Model and Particle Swarm Optimization Algorithm Based on the Model-Optimization Method for Improving Soil Moisture Simulation in a Semi-Arid Region

**DOI:** 10.1371/journal.pone.0151576

**Published:** 2016-03-18

**Authors:** Qidong Yang, Hongchao Zuo, Weidong Li

**Affiliations:** 1 Department of Atmospheric Sciences, Yunnan University, Kunming, Yunnan Province, China; 2 College of Atmospheric Sciences, Lanzhou University, Lanzhou, Gansu Province, China; Southwest University, CHINA

## Abstract

Improving the capability of land-surface process models to simulate soil moisture assists in better understanding the atmosphere-land interaction. In semi-arid regions, due to limited near-surface observational data and large errors in large-scale parameters obtained by the remote sensing method, there exist uncertainties in land surface parameters, which can cause large offsets between the simulated results of land-surface process models and the observational data for the soil moisture. In this study, observational data from the Semi-Arid Climate Observatory and Laboratory (SACOL) station in the semi-arid loess plateau of China were divided into three datasets: summer, autumn, and summer-autumn. By combing the particle swarm optimization (PSO) algorithm and the land-surface process model SHAW (Simultaneous Heat and Water), the soil and vegetation parameters that are related to the soil moisture but difficult to obtain by observations are optimized using three datasets. On this basis, the SHAW model was run with the optimized parameters to simulate the characteristics of the land-surface process in the semi-arid loess plateau. Simultaneously, the default SHAW model was run with the same atmospheric forcing as a comparison test. Simulation results revealed the following: parameters optimized by the particle swarm optimization algorithm in all simulation tests improved simulations of the soil moisture and latent heat flux; differences between simulated results and observational data are clearly reduced, but simulation tests involving the adoption of optimized parameters cannot simultaneously improve the simulation results for the net radiation, sensible heat flux, and soil temperature. Optimized soil and vegetation parameters based on different datasets have the same order of magnitude but are not identical; soil parameters only vary to a small degree, but the variation range of vegetation parameters is large.

## Introduction

Soil moisture is an important component of global energy and water circulation. The soil moisture can directly affect meteorological and hydrological processes by affecting physical processes such as surface evaporation, vegetation transpiration, and runoffs [1,2,3,4] and can indirectly affect the global carbon circulation by affecting vegetation growth and plant photosynthesis [5,6,7,8,9]. Thus, accurately observing and simulating the soil moisture is critical for studying global climate change [10,11]. However, the spatial and temporal inhomogeneity of the soil moisture distribution causes great difficulties in its observation and simulation. On one hand, although techniques such as time domain reflectometry (TDR) can be used to observe the soil moisture at stations [12], it is difficult to obtain high-precision, large-scale, and long-term observational data. On the other hand, although extant land-surface process models or climate models can be used to simulate long-term variation trends of the soil moisture, there exist large offsets between simulated results and observational data [13]. Furthermore, different results are obtained by using different models [14,15,16]; consequently, it is difficult to establish reliable global soil moisture datasets through model simulations. Thus, it is of significant importance to improve the capability of land-surface models to simulate the soil moisture and thereby improve numerical weather forecast and climate predictions.

Generally, the simulation capability of a land-surface process model is closely related to the parameterization schemes and input parameters of the model [14]. The parameterization schemes adopted in a model is built based on field observational data; therefore, different models adopt different parameterization schemes for plants and soil. Comparisons of multiple land-surface process models have indicated that different land-surface parameterization schemes have a significant impact on the simulation results [15,17,18]. Based on the actual land-surface condition, an appropriate parameterization schemes can improve the simulation capability. In addition, land-surface process models require the input of multiple parameters for simulation, including vegetation and soil parameters, terrain parameters, and the initial soil hydrothermal conditions. These parameters significantly affect the simulation results [19,20,21]. Some of these parameters can be obtained with high precisions by station or remote-sensing observations. For instance, the soil content, vegetation root distribution, surface aerodynamic roughness, and initial soil temperature can be obtained by station observation. By contrast, the vegetation leaf area index, vegetation height, and surface albedo can be measured by large-scale remote sensing. However, it is difficult (if not impossible) to observe some parameters for the following reasons: (1) Values obtained by station observation cannot represent values on a large scale. Because certain parameters, such as the saturated soil water conductivity, vary at different locations, the observed value at one point cannot be used for its neighboring point; (2) Some parameters can be interactively connected, and it is difficult to precisely measure all of them together, for example, the empirical parameter of vegetation transpiration stomatal resistance; (3) Some parameters used in a model do not have definitive physical meaning and therefore cannot be observed, for example, the Clapp-Hornberger constant. To overcome these difficulties, different combinations of land-surface parameters are used in a model, and by comparing differences between simulation results and observational data, the adaptability of model parameters can be evaluated, which is known as the parameter calibration process. Multiple studies have demonstrated that the simulation capability of land-surface process models can be improved by calibrating adopted parameters [22,23].

In the past 20 years, intelligent or optimized algorithms have attracted wide interest with respect to calibrating land-surface models. For instance, the SCE(Shuffled Complex Evolution) global optimization method has been used to calibrate the hydrological model [24]; Gupta et al. adopted multicriteria methods for parameter estimation, which (1) proves effective when only the range of parameter physical values is known and (2) can improve the simulation capability of the BATS(Biosphere-Atmosphere Transfer Scheme) model [25]. In addition, to assimilate the soil moisture, Ines et al. also used the genetic algorithm to estimate hydrological parameters [26]. In recent years, another optimization algorithm, particle swarm optimization, has become popular [27] and has been widely applied in other research areas [28]. This algorithm was built on the basis of animal behaviors, such as the process of searching for food by fish or birds, which essentially is a particle constrained by a certain object function solving a global (or approximately global) optimal solution. Calibrating parameters in land-surface process models is a similar process, i.e., choosing different parameter combination methods to reduce the difference between observational and simulation results during a certain period. Thus, many researchers have used particle swarm optimization to optimize parameters for hydrological models. For example, Gill et al. used the multiple-objective particle swarm optimization to estimate hydrological parameters [29]; Chaw et al. used the particle swarm optimization method to predict the water level by combining ANNs(Artificial Neural Networks)[30]; Scheerlinck compared the similarity and difference in optimizing model parameters between the MWAPRE(Multistart Weight-adaptive Recursive Parameter Estimation) and particle swarm optimization algorithms and found that the particle swarm optimization algorithm is more practical and more effective in utilizing observational data [31]; Zhang et al. evaluated the pros and cons of five optimization methods in calibrating hydrological models [32] and found that compared with other methods, particle swarm optimization can be used to obtain the optimal parameter solution, which also takes less time.

The semi-arid region is approximately 40% of the global land surface [33]. Its surface types are mainly composed of sparse vegetation, grassland, and desert, whose surface characteristics significantly differ from that of the humid region [34]. In addition, the semi-arid region is sensitive to climate change and has the highest variability in precipitation [35]. Therefore, the ecological and water-resource systems in the semi-arid region are closely related to the soil moisture [9,36]. Vegetation destruction and grassland desertification caused by human activities can further cause an anomalous change in the soil moisture, resulting in negative feedback of the climate system and consequently threatening the human living environment [9,36,37,38]. Due to the lack of knowledge regarding the specific characteristics of the land-surface process in semi-arid regions, limited near-surface observational experiments, and large offsets in large-scale parameters obtained by the remote-sensing method, land-surface process models have a low simulation capability [39]. Thus, conducting comprehensive near-surface observation experiments, accurately identifying land-surface parameters or parameter combinations using optimized methods are critical for improving the soil moisture simulation capability in semi-arid regions. However, several limitations and difficulties still exist in former studies. (1) A complete land surface process model contains the vegetation, soil, snow and atmospheric boundary layer, involving many parameters which are dependent. But in the existing study, most of the optimization algorithms only used for simple or simplified hydrological model [26,29,31], therefore, the optimized parameters can not be applied in complete models. (2) In previous studies, the optimization parameters were selected arbitrarily and the related physical processes were not considered [25]. The dimension of the optimization parameters space was too high and the parameters combinations were too many. Hence, the optimized parameters combinations cannot be used in models. For example, all the input parameters were selected for optimization. (3) In previous studies, optimized parameters were usually obtained by a single and short length dataset. Correspondingly, the optimized parameters are not accurate for longtime simulation.

To address the aforementioned issues, this study utilizes the meteorological data from the Semi-Arid Climate Observatory and Laboratory station in the semi-arid loess plateau region of China and divided the data into different datasets. By adopting the particle swarm optimization method, this study optimizes soil and vegetation parameters related to soil moisture in the land-surface process model, SHAW (Simultaneous Heat and Water). On this basis, the optimized parameters are utilized in the SHAW model to improve the capability of the SHAW model to simulate the soil moisture in the semi-arid region.

## Methodology

### SHAW Model

The SHAW model was developed by Flerchinger et al [40,41] and was initially used to simulate the freezing and melting of soil. After continuous development and improvement, SHAW gradually forms a comprehensive land-surface model, which includes interactions between soil, the accumulated snow-residue layer, vegetation, and atmosphere. The SHAW model can divide the vegetation and residue into less than 10 layers, the accumulated snow into less than 100 layers, and the soil into less than 50 layers. In addition, the model considers radiation transfer, convective exchange, hydrothermal transport in soil, precipitation infiltration, and soil freeze-up and melt between different physical layers. The SHAW model is a single-point land surface model, most of the vegetation and soil parameters can be directly observed based on the actual underlying conditions. Only a few of input parameters in the SHAW model which need optimize are difficult to observe. The dimension of optimized parameters space is low. Most of the input parameters in SHAW model can be easily transferred to other land surface models. The model has been applied to simulation studies on different underlying surfaces. A series of tests have been conducted for complicated underlying surfaces in the semi-arid region [40,42].

The controlling equation of the soil moisture in the SHAW model can be expressed as [43]:

$$\frac{\partial \theta_{l}}{\partial t}+\frac{\rho_{i}}{\rho_{l}}\frac{\partial \theta_{i}}{\partial t}=\frac{\partial }{\partial z}[K(\frac{\partial \psi }{\partial z}+1)]+\frac{1}{\rho_{l}}\frac{\partial q_{v}}{\partial z}+U$$ (1)

in which, *z* represents the soil depth (m), *t* represents time (s), *θ*_*l*_ (*θ*_*i*_) represents the soil volumetric water (ice) content (m^3^ m^-3^), *ρ*_*l*_ (*ρ*_*i*_) represents the water (ice) density (kg m^-3^), *K* represents the water conductivity (m s^-1^), *ψ* represents the soil water potential (m), *q*_*v*_ represents the soil water vapor density (kg m s^-1^) (which is determined by the soil volumetric water content), and *U* represents the vegetation root water absorption (m^3^ m^-3^ s^-1^).

The soil water potential *ψ* and water conductivity can be calculated using the following equation:

$$\psi =\psi_{e}{\left(\frac{\theta_{l}}{\theta_{s}}\right)}^{-b}$$ (2)

$$K=K_{s}{\left(\frac{\theta_{l}}{\theta_{s}}\right)}^{\left(2b+3\right)}$$ (3)

in which *ψ*_*e*_ is the air entry potential (m), *b* is the Clapp-Hornberger constant, *θ*_*s*_ is the saturated water content (m^3^ m^-3^), and *K*_*s*_ is the water conductivity when the soil is saturated (m s^-1^).

The vegetation root water absorption *U* depends mainly on the vegetation transpiration, which is determined by the soil-vegetation-air water transport and can be expressed as:

$$T_{j}=\sum_{k=1}^{NS}\frac{\psi_{k}-\psi_{x,j}}{r_{r,j,k}}=\sum_{i=1}^{NC}\frac{\psi_{x,j}-\psi_{l,i,j}}{r_{l,i,j}}=\sum_{i=1}^{NC}\frac{\rho_{vs,i,j}-\rho_{v,i}}{r_{s,i,j}+r_{h,i,j}}L_{i,j}$$ (4)

in which *i* is the canopy, *j* is the number of plant species, *k* is the soil layer, *NC* is the total number of the canopy layers, *NS* is the total number of the soil layers, *T*_*j*_ is the total transpiration (kg m^-2^ s^-1^), *L*_*i*,*j*_ is the leaf surface area index, *ρ*_*vs*,*i*,*j*_ and *ρ*_*v*,*i*_ are the water vapor densities of the leaf surface and of the air inside the canopy, respectively (kg m^-3^), *ψ*_*x*,*j*_ and *ψ*_*l*,*i*,*j*_ are the water potentials of the vegetation xylem and of the leaf, respectively (m), *r*_*h*_ and *r*_*s*_ are canopy air and stomatal resistances, respectively, (s m^-1^), and *r*_*l*_ and *r*_*r*_ are leaf and root resistances, respectively (m^3^s kg^-1^), which can be expressed as:

$$r_{h}=307{\left(d_{l}/u\right)}^{1/2}$$ (5)

$$r_{s}=r_{so}[1+{\left(\psi_{l}/\psi_{c}\right)}^{5}]$$ (6)

$$r_{l}=r_{lo}(L_{i}/L)$$ (7)

$$r_{r}=r_{ro}(D_{p,i}/D_{p})$$ (8)

in which *d*_*l*_ is the Characteristic dimension of the leaves(m), *u* is the wind speed within the canopy (m s^-1^), *r*_*so*_ is the minimal stomatal resistance (s m^-1^), *r*_*lo*_ and *r*_*ro*_ are the leaf and root resistance constants, respectively (m^3^s kg^-1^), *ψ*_*c*_ is the critical leaf water potential, and *L*(*L*_*i*_) and *D*_*p*_(*D*_*p*,*i*_) are the total leaf surface area (the leaf surface area of each layer) and total root ratio (the root ratio of each layer), respectively.

For the soil-moisture controlling equation described above, its upper boundary condition can be expressed as:

$$q_{s}=P-E_{s}-R_{s}$$ (9)

in which *q*_*s*_ is the water flux that enters the soil (m s^-1^), *P* is the precipitation rate or melting rate of accumulated snow (m s^-1^), *E*_*s*_ is the evaporation of the soil surface (m s^-1^), and *R*_*s*_ represents surface runoffs (m s^-1^).

In the lower boundary condition of the soil moisture, the gradient of the soil moisture is set at zero. Finally, land-surface process models must also satisfy the energy balance equation, which is:

$$R_{n}=H+L_{v}E+G$$ (10)

in which *G* is the soil heat flux (W m^-2^), *R*_*n*_ is the net radiation (W m^-2^), *H* is the sensible heat flux (W m^-2^), *E* is the water vapor flux (kg m^-2^ s^-1^), and *L*_*v*_ is the potential evaporation coefficient. The method above used to parameterize factors related to the soil moisture shows that all of the vegetation and soil parameters (including *ψ*_*e*_, *b*, *θ*_*s*_, *K*_*s*_, *r*_*so*_, *r*_*lo*_, *r*_*ro*_, *ψ*_*c*_, *d*_*l*_, *L*, and *D*_*p*_) can affect simulations of the soil moisture.

### PSO Algorithm

The PSO algorithm was first introduced by Kennedy et al.[44] to simulate the society physiological behavior and was later expanded to other applications and became an optimization method to solve the global optimal solution for large-scale non-linear problems. The principle of PSO is to assign coordinates and initial velocities for a group of randomly chosen particles and then search in the space within a defined region. By continuously updating the positions and velocities of these particles, the algorithm compares the object function of each particle to obtain the local optimal position and finally the global optimal position.

If we want to optimize an *n*-dimensional problem for *m* particles, the position and velocity vector of the *i*^th^ particle can be expressed as:

$$x_{i}=(x_{i1},x_{i2},\ldots ,x_{in})$$ (11)

$$v_{i}=(v_{i1},v_{i2},\ldots ,v_{in})$$ (12)

The updated position and velocity of the i^th^ particle can be expressed as:

$$v_{in}^{N+1}=\omega v_{in}^{N}+c_{1}r_{1}(p_{in}^{N}-x_{in}^{N})+c_{2}r_{2}(G_{n}^{N}-x_{in}^{N})$$ (13)

$$x_{in}^{N+1}=x_{in}^{N}-v_{in}^{N}$$ (14)

in which *N* represents the number of iterations; *w* represents the inertia weight; *c*_*1*_ and *c*_*2*_ are the acceleration constants, which are the weight coefficients of the optimal value by tracking its own history and therefore represent self-awareness of the particle; *r*_*1*_ and *r*_*2*_ are random numbers in [0,1]. ***p***_*i*_ and ***G***_*n*_ represent the optimal value of the *i*^th^ particle by searching its history and the optimal position searched by all the particles, respectively, which can be expressed as:

$$p_{i}=(p_{i1},p_{i2},\ldots ,p_{in})$$ (15)

$$G_{n}=(p_{g1},p_{g2},\ldots ,p_{gn})$$ (16)

$$g=\underset{1\leq i\leq n}{\text{min}}[f(p_{i})]$$ (17)

in which *g* represents the position when the value of the object function is the lowest and **f** is the object function. The object function **f** in the PSO algorithm can be a single function or vector function. When **f** is a vector function, it should be the multiple object function; therefore, one method is to solve for its Pareto front, and another method, proposed by Crow et al.[45], is to standardize multiple variables with different orders of magnitude and then define a single object function to solve for its minimum.

## Data and Method

### Data

Data used in this study is from the Semi-Arid Climate Observatory and Laboratory (SACOL) station located in Yuzhong County in Gansu Province (35.946°N, 104.137°E). The SACOL station is within the loess plateau region in China, which can represent the climate condition within a few hundreds of kilometers in the semi-arid region [46]. Its altitude is 1,961 m, and the underlying surface is flat with short grass growing. The SACOL station is equipped with a micro meteorological tower, three dimension (3D) sonic anemographs, temperature and moisture detector, and soil monitoring system et al. The details can be found at (http://climate.lzu.edu.cn/english/index.asp). The observation data have been widely applied to studies on the semi-arid climate and regional energy and water circulation. The SACOL station is included in the CEOP (Coordinated Enhanced Observing Period), AERONET (Aerosol Robotic Network), and MPLNET (Micro-Pulse Lidar Network) international meteorological observational websites, and the observational data are opened for any researchers who were interested in climate change in the semi-arid region in Northwest China. The filed area was not protected and filed studies did not involve endangered or protected species.

### Model-optimization Method

**Fig 1:**
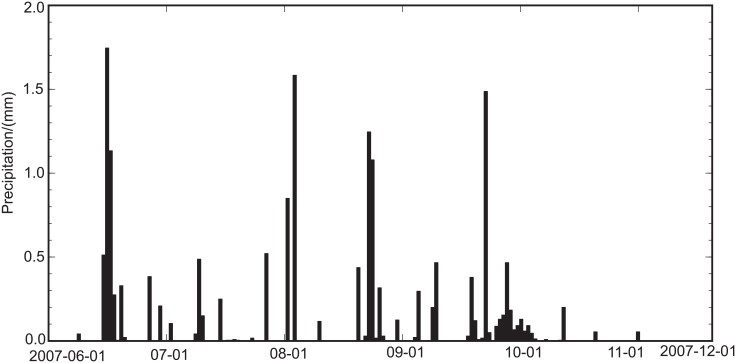
Precipitation during the simulation period.

To run land-surface process models, input variables include the atmospheric forcing condition, the initial condition for the soil temperature and moisture, and surface vegetation and soil parameters. In this study, the simulation period is set to the summer and fall seasons of 2007 (from 01/06 to 30/11) to avoid the effect of snow. According to the actual underlying surface condition, we divide the vegetation into one layer and the soil into six layers of 5, 10, 20, 40, 80, and 250 m, which is consistent with the observational depths. The atmospheric forcing variable adopts the hourly wind speed, temperature, pressure, precipitation, relative humidity, downward short-wavelength radiation, and downward long-wavelength radiation at the SACOL station. The soil moisture is mainly affected by the precipitation; therefore precipitation during the simulation period is show in Fig 1.

The initial conditions of the soil temperature and moisture are taken from the actual observational data on 01/06/2007. If a surface parameter can be directly observed, then the observational value is adopted in the model; if a parameter is difficult to observe, it is then solved for its optimal value or parameter combination using the particle swarm algorithm. According to the parameterization schemes for the soil moisture in the SHAW model discussed above, we chose *ψ*_*e*_, *b*, *θ*_*s*_, *K*_*s*_, *r*_*so*_, *r*_*lo*_, *r*_*ro*_, and *ψ*_*c*_ as the parameters for optimization. Because the soil layer is divided into six layers, there are 16 parameters in total by assuming that the *ψ*_*e*_, *b*, *θ*_*s*_, *K*_*s*_ values of adjacent layers are the same.

When utilizing the PSO algorithm to optimize parameters, an object function must be defined. In this study, the Kling-Gupta efficiency (KGE) function proposed by Gupta et al is used as the object function, which is defined as [47]:

$$KGE=1-\sqrt{{\left(r-1\right)}^{2}+{\left(\alpha -1\right)}^{2}+{\left(\beta -1\right)}^{2}}$$ (18)

in which *r* represents the correlation coefficient between the observational and simulation value, *α* represents the ratio of the standard deviation of the observational value to that of the simulation value, and *β* represents the ratio of the mean observational value to the mean simulation value. KGE is used to evaluate the quality of the fit for the simulation result with the observation, whose range varies from -∞ to 1; the closer the value is to 1, the better the simulation capability. Corresponding *KGE*_*j*_ (j represents the corresponding soil moisture, temperature, etc.) values are calculated using the soil moisture, surface temperature, sensible heat flux, latent heat flux, net radiation, and corresponding observational values of the five-layers soil simulated by the SHAW model. The final KGE is the average of all *KGE*_*j*_ values. Because all these variables have different orders of magnitude, they are standardized during the calculation of both simulation and observation, i.e., the corresponding average value is subtracted from each observational or simulation value, which is then divided by the corresponding standard deviation [31].

**Fig 2:**
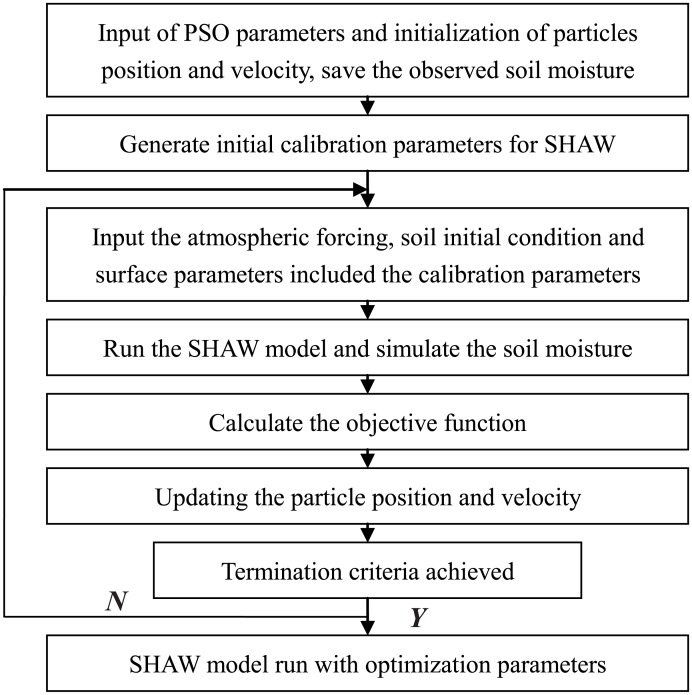
SHAW-PSO method flow chart.

The PSO algorithm also depends on parameters of the model itself, specifically, the number of particle swarm, *N*, *c*_*1*_, c_2_, w, and the position and velocity variation range of each particle. According to multiple simulation tests and previous studies [29,31], (1) n = 20; (2) *N* = 300; (3) the variation range of w is from 0.2 to 0.5; (4) *c*_*1*_ = 1.7, and *c*_*2*_ = 2; (5) the variation range of the particle position is from -1 to 1, and that of the particle velocity is from -0.01 to 0.01. For all parameters that must be optimized, their variation ranges become [–1,1] by the following method:

$$x=\frac{2y-(R_{\text{max}}+R_{\text{min}})}{\left(R_{\text{max}}-R_{\text{min}}\right)}$$ (19)

in which **y** is the actual value of a parameter for optimization and *R*_*max*_ and *R*_*min*_ represent the range of the parameter for optimization. Based on the process described above, the combination of the SHAW model and the PSO algorithm is called the SHAW_PSO method. The detailed realization method is depicted in the following flow chart (Fig 2).

To optimize parameters, the datasets are divided into three groups: the first group consists of summer (June-August) data, the second group consists of autumn (September-November) data, and the third group consists of data from the summer-autumn (June-November) period, which are used for separate parameter optimization. The results of the SHAW model obtained by adopting the optimized parameters are tested against all the summer and autumn data, which are recorded as “SHAW_PSO_SU”, “SHAW_PSO_AU”, and “SHAW_PSO_SA”. As a control test, the model was also run with the same atmospheric forcing variables, the initial condition, and the default parameters of the model given in Table 1, named “SHAW_DEFAULT”. In Table 1, the leaf area index is evaluated by the NDVI (Normalised Difference Vegetation Index) which is measured by MODIS (Moderate Resolution Imaging Spectroradiometer) satellite. The retrieval method given by reference is employed to calculate the leaf area index [48]. The plant height, Characteristic dimension of the leaves and effective rooting depth is measured by ruler, and the mean value is used. The percent of sand, silt, and clay is measured in soil laboratory. Dry soil albedo is evaluated by the downward and upward shortwave radiation. The aerodynamic roughness is estimated by wind speed at different height. For all parameters that must be optimized, the thresholds of these parameters are also listed in Table 1. The thresholds of soil parameters are primarily based on previous studies [49]. The thresholds of vegetation parameters are difficult to evaluate, hence 0.1 or 10 times of the model suggest values are set as the range.

**Table 1:** Input parameters for the SHAW-PSO model.

Variable	Symbol	Default	Unit	Range
***Vegetation parameters***				
Plant albedo	*α*_*c*_	0.23	--	--
Transpiration temperature	*T*_*c*_	7	K	--
Minimum stomatal resistance	*r*_*so*_	100	m s^-1^	[10,1000]
Critical leaf water potential	*ψ*_*c*_	-100	m	[-10,-1000]
Leaf resistance	*r*_*lo*_	1e5	m^3^ s kg^-1^	[1e4,1e6]
Root resistance	*r*_*ro*_	2e5	m^3^ s kg^-1^	[2e4,2e6]
Plant height	*H*	0.15	m	--
Characteristic dimension of the leaves	*d*_*l*_	5e-3	m	--
Dry biomass	*W*_*g*_	0.5	kg m^-2^	--
Leaf area index	*L*	1.5	--	--
Effective rooting depth	*D*_*p*_	0.15	m	--
***Soil parameters***				
Air-entry potential	*ψ*_*e*_	-0.31	m	[-1.0,-0.1]
Campbell’s pore-size index	*b*	4.5	--	[3,10]
Saturated conductivity	*K*_*s*_	2e-6	m s^-1^	[5e-5,5e-7]
Saturated volumetric moisture content	*θ*_*s*_	0.43	--	[0.3,0.6]
Bulk density	*ρ*_*b*_	1020	kg m^-3^	--
Sand percent	*sand%*	38	*%*	--
Silt percent	*silt%*	26	*%*	--
Clay percent	*clay%*	22	*%*	--
Organic percent	*om%*	14	*%*	--
Dry soil albedo	*α*_*s*_	0.30	--	--
Exponent for the calculated albedo	*a*	-2	--	--
Aerodynamic roughness	*z*_*om*_	0.46	--	--

## Results

**Fig 3:**
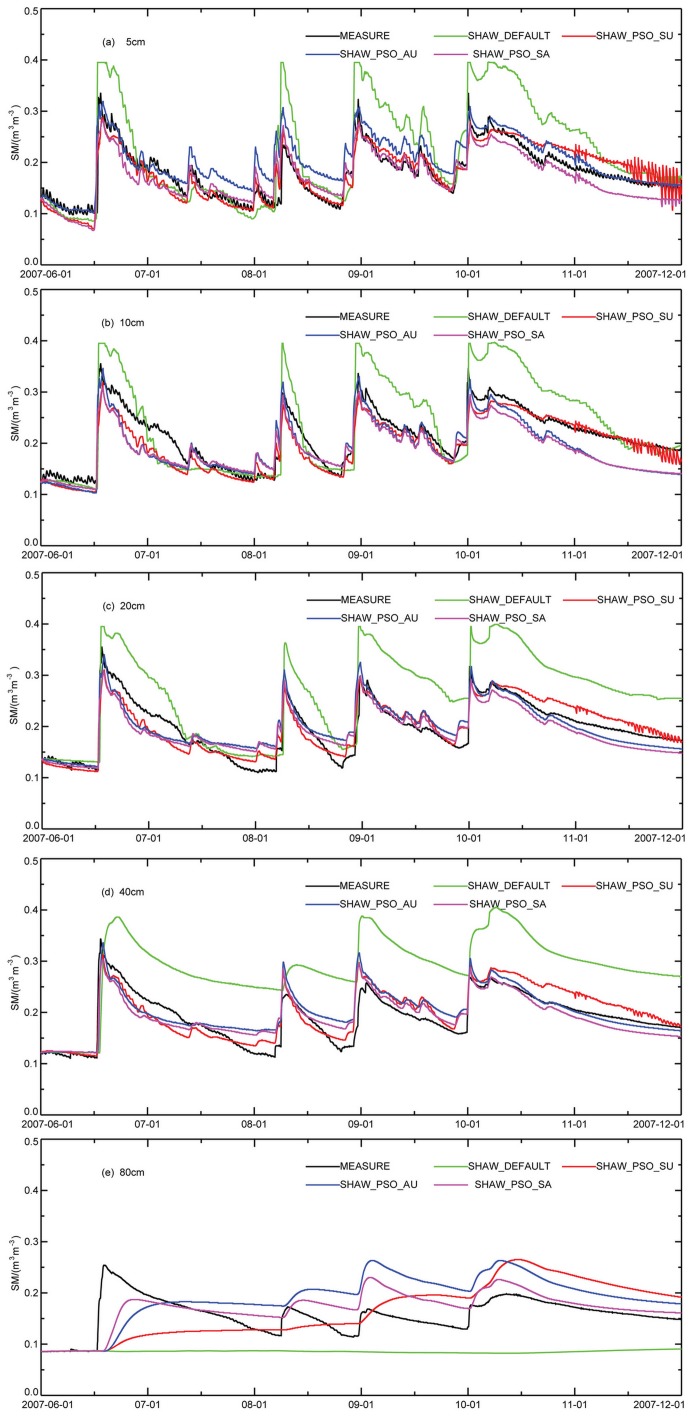
Comparison of the soil moistures calculated by different sets of simulation tests with the observational values. (a) 5cm (b) 10cm (c)20cm (d)40cm (e)80cm.

Fig 3a–3e shows the comparison of soil moisture (SM) values simulated by the four sets of simulation tests with the observational data. As presented in the Fig 3, all the simulation results after parameter optimization can reasonably reproduce the variation trend of the soil moisture, but the SHAW_DEFAULT test cannot predict the variation trend below 40 cm, indicating that the soil and vegetation parameters suggested by the model differ from the actual underlying surface. Table 2 lists the root-mean-square deviation and the KGE value of the simulated results with respect to the measured data, which clearly illustrate that offsets of the simulation tests are all reduced after parameter optimization relative to that of the SHAW_DEFAULT test; their corresponding KGE values are also increased, which demonstrates that parameter optimization significantly improves the model’s simulation capability. By comparing the three sets of simulation tests after parameter optimization, we can observe that the soil moistures of all the layers above 80 cm simulated by SHAW_PSO_SU are closer to the measured data than are the values from the other two simulation tests. The deep soil moisture is related to the underground water. The SHAW model does not contain the underground water parameterization scheme. Hence, the absence of the underground water parameterization may also affect the soil moisture simulation.

**Table 2:** Root mean square error and the KGE value of the simulated soil moisture at different depths.

Depth (cm)	MEAN OBS	SHAW_DEFAULT			SHAW_PSO_SU			SHAW_PSO_AU			SHAW_PSO_SA		
		MEAN	RMBE	KGE	MEAN	RMBE	KGE	MEAN	RMBE	KGE	MEAN	RMBE	KGE
**5cm**	0.179	0.221	0.067	0.48	0.181	0.022	0.89	0.201	0.031	0.84	0.169	0.024	0.86
**10cm**	0.211	0.233	0.049	0.58	0.196	0.023	0.89	0.191	0.032	0.83	0.187	0.035	0.71
**20cm**	0.196	0.260	0.075	0.55	0.198	0.021	0.90	0.199	0.025	0.79	0.189	0.026	0.70
**40cm**	0.191	0.286	0.104	0.50	0.199	0.023	0.88	0.200	0.026	0.82	0.191	0.024	0.77
**60cm**	0.185	0.178	0.078	0.12	0.193	0.028	0.80	0.206	0.038	0.69	0.181	0.028	0.65
**80cm**	0.157	0.085	0.079	-15.4	0.164	0.054	0.22	0.189	0.057	0.25	0.169	0.035	0.52

**Fig 4:**
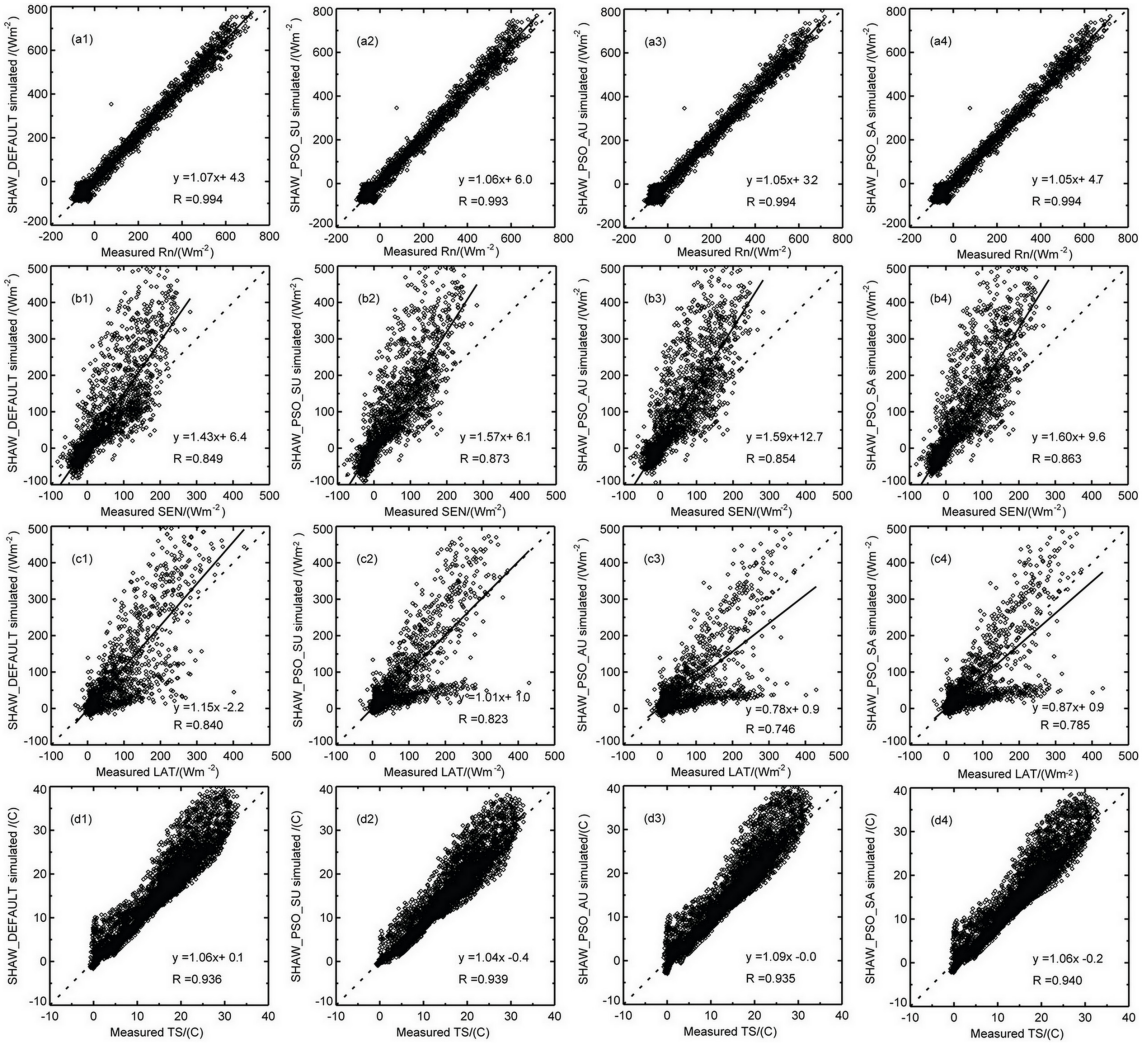
Scatter plots of the net radiation, sensible and latent heat fluxes, and soil temperature relative to the corresponding observational data in different sets of simulation tests. (a1-a4) Radiation (b1-b4) Sensible heat flux (c1-c4) Latent heat flux (d1-d4) Soil temperature at 5cm depth.

Fig 4 shows the scatter plots of the net radiation, sensible heat and latent heat fluxes, and soil temperature at 5 cm calculated by different sets of simulation tests, compared with the corresponding observational data. Table 3 lists the corresponding offsets, average values, and KGE values of different simulation tests. As shown in the figure, the simulations of net radiation(Fig 4a1–4a4) produced by different sets of model tests are all close to the observational data, which can be demonstrated by the close to 1:1 linear fit lines with correlation coefficients all above 0.99. The offsets given in Table 3 indicate that the net radiation simulated by SHAW_PSO_AU is the closest to the observational data with the highest KGE. The correlation coefficients between the simulated sensible heat flux values (Fig 4b1–4b4) and the observational data are all high (> 0.85), but all of the simulated values are higher than the observational data. Table 4 shows that the offset between the sensible heat flux and the observational data is the smallest for the simulation by SHAW_DEFAULT. The correlation coefficients of the latent heat flux (Fig 4c1–4c4) values simulated by different sets of simulation tests and the observational value are all above 0.75, and the model tests with optimized parameters all have improved simulation results for the latent heat flux relative to SHAW_DEFAULT. In particular, the latent heat flux simulated by SHAW_PSO_SA agrees the best with observation and has the highest KGE. The correlation coefficients between the simulation results of different model tests and the observational data for the soil temperature (Fig 4d1–4d4) are all above 0.94, and the linear fit lines are close to the 1:1 line; the soil temperature simulated by SHAW_PSO_SU is most consistent with the observation. Based on analysis of the net flux, sensible heat and latent heat fluxes, and soil temperature, we can observe that the simulation capabilities for the latent heat flux in different tests are all improved, but the simulation results for all of the subcomponents cannot be improved simultaneously. This finding is similar to the Pareto front yielded by the multiple object function method [22]. Previous studies by Gupta et al have also indicated that the adoption of optimized parameters cannot improve the simulation capabilities for all the variables in land-surface process models [25]. In addition, the large offsets of simulation results for the sensible and latent heat fluxes are perhaps related to the energy closure degree in this region. Previous relevant studies have demonstrated that the average energy closure degree in this region is approximately 0.75 [50]. In this study, the energy closure in summer and autumn is 0.78 and 0.77. Because the SHAW model is built on the basis of the energy closure, it necessarily results in offsets in the simulation results relative to the observational data, thereby revealing that the simulation capability of a model is related to the model structure, the parameterization schemes adopted in the model, and the energy closure degree during observation.

**Table 3:** Root mean square error and the KGE value of the simulated net radiation, sensible, latent heat flux and soil temperature.

Variable	MEAN OBS	SHAW_DEFAULT			SHAW_PSO_SU			SHAW_PSO_AU			SHAW_PSO_SA		
		MEAN	RMBE	KGE	MEAN	RMBE	KGE	MEAN	RMBE	KGE	MEAN	RMBE	KGE
**Rn**	99.17	108.35	25.11	0.89	108.47	25.51	0.89	105.65	21.43	0.92	106.37	22.25	0.91
**Sen**	22.63	38.44	59.39	0.36	41.92	63.26	0.32	47.34	70.28	0.25	45.26	68.52	0.27
**Lat**	41.49	47.01	51.38	0.65	46.27	47.61	0.71	34.62	47.78	0.67	39.52	46.11	0.75
**Tg**	15.27	16.36	3.37	0.84	15.44	3.00	0.89	16.62	3.58	0.82	15.96	3.15	0.86

**Table 4:** Parameters optimized based upon different datasets.

variables	*r*_*so*_	*ψ*_*c*_	*r*_*lo*_	*r*_*ro*_	*ψ*_*e*,*1*_	*ψ*_*e*,*2*_	*ψ*_*e*,*3*_	*θ*_*s*,*1*_	*θ*_*s*,*2*_	*θ*_*s*,*3*_	*K*_*s*,*1*_	*K*_*s*,*2*_	*K*_*s*,*3*_	*b*_*1*_	*b*_*2*_	*b*_*3*_
	m s^-1^	m	m^3^ s kg^-1^	m^3^ s kg^-1^	m	m	m	m^3^ m^-3^	m^3^ m^-3^	m^3^ m^-3^	m s^-1^	m s^-1^	m s^-1^	--	--	--
**SHAW_PSO_SU**	205	-365	6.85e5	1.49e6	-0.58	-0.49	-0.15	0.37	0.35	0.43	2.86e-6	3.63e-6	2.68e-6	4.82	4.72	6.22
**SHAW_PSO_AU**	401	-268	3.45e5	1.64e6	-0.92	-0.68	-0.62	0.40	0.39	0.37	2.50e-6	3.43e-6	4.08e-6	6.31	7.92	5.98
**SHAW_PSO_SA**	328	-368	1.62e5	1.30e6	-0.56	-0.55	-0.51	0.37	0.41	0.40	4.04e-6	4.17e-6	2.77e-6	5.18	4.85	5.85

Table 4 summarizes parameters optimized based upon different datasets. The table reveals that the different sets of parameters are not identical, whereas they all have the same order of magnitude. The parameters related to soil (*ψ*_*e*_, *b*, *θ*_*s*_, and *K*_*s*_) optimized by using different datasets are consistent with small variation ranges, whereas the parameters related to vegetation (*r*_*so*_, *r*_*lo*_, *r*_*ro*_ and *ψ*_*c*_) all vary to large degrees, which might be related to the underlying surface condition: the soil parameters only slightly vary with changing seasons, whereas the vegetation parameters are strongly dependent on seasons. Thus, in land-surface process models, it is more appropriate to set the vegetation parameters as time-dependent variables.

## Conclusions and Discussion

Soil moisture is an important component in energy and water circulations. For simulation studies on climate, it is crucial to accurately simulate soil moisture. However, there still exist large offsets in current land-surface process models, which are coupled with climate models via land-surface process models and therefore cause uncertainties in the simulation results of weather and climate models. Thus, it is critical to accurately observe and simulate soil moisture. The semi-arid region is a belt that is sensitive to climate change, and variation in the soil moisture is of great importance to the regional climate. Because of the lack of knowledge regarding the specialty of the land-surface process in semi-arid regions, limited near-surface observational experiments, and large offsets in large-scale parameters obtained by the remote sensing method, there are large deviations of the simulated soil moisture from the observational data. To improve the capability of land-surface process models to simulate soil moisture in semi-arid regions, we adopted the PSO algorithm and data from the SACOL station in the semi-arid loess plateau to be used for comparison with the simulation results of the SHAW model with optimized soil and vegetation parameters and obtained following conclusions:

1. Different simulation tests of the SHAW model optimized by the PSO algorithm based on different datasets can all significantly improve simulations for the soil moisture and latent heat flux. In particular, the SHAW_PSO_SU model results agree the best with the observational data for soil moisture above 80 cm (with the highest KGE value), whereas the latent heat flux simulated by the SHAW_PSO_SA model shows minimal deviation from the observational data (with the highest KGE value). These improvements indicate that after optimization of parameters related to soil moisture, the simulation capability of the SHAW model for soil moisture and latent heat flux is improved.
2. The optimized SHAW model cannot well simulate the net radiation, sensible heat flux, and soil temperature simultaneously. In particular, the net radiation simulated by SHAW_PSO_AU shows the smallest offset from the observational data, which also has the highest KGE value. In addition, the sensible heat flux calculated by SHAW_DEFAULT agrees the best with observation and has the highest KGE value. Finally, the soil temperature simulated by SHAW_PSO_SU is most consistent with observation and has the highest KGE value. These results indicate that the simulation capability of a model is not only related to input parameters but also depends strongly on the model structure, the parameterization schemes, and the energy closure degree during observation.
3. The soil and vegetation parameters are not identical among optimizations based on different datasets, but all have the same order of magnitude. The varying range of parameters related to soil is limited, whereas that related to vegetation is large, which might be associated with the characteristics of the underlying surface. For instance, soil parameters vary with the season to a small degree, whereas vegetation parameters significantly change with seasons. Thus, it is more appropriate to set vegetation parameters as time-dependent variables in land-surface process models.

Our study showed that the SHAW model, by adopting parameters related to the soil moisture optimized by the PSO algorithm, can improve the simulation capability for soil moisture. In simulation studies, there still exist a few problems in using the PSO algorithm or other optimization algorithms, which must be addressed in future studies, as follows: (1) Parameters obtained by an optimization algorithm should be further tested against observations. Although optimized parameters or parameter combinations can improve the simulation capability of land-surface process models, some of these parameters have specific physical meanings. Thus, optimized parameters must satisfy their corresponding physical variation ranges and therefore cannot be only mathematically treated as the optimal solutions; (2) The dimension of a parameter for optimization cannot be too high. There is a limit; in fact, the higher the dimension is, the more corresponding combination methods for the parameters and, in turn, the larger the variation ranges of the optimized parameters. Thus, in practice, observation and optimization algorithms should be complementarily combined: if a parameter can be observed, the observational value should be used; for parameters that are difficult to observe, an optimization algorithm should be adopted; (3) The simulation test results in this study showed that soil moisture simulated by adopting the summer dataset is optimal, whereas the results based on the summer-autumn datasets is not the optimal. Thus, it appears that parameter optimization is irrelevant to the length of the dataset. Further studies on how to appropriately choose a dataset for parameter optimization are therefore required. In summary, it is of equal importance to conduct comprehensive near-surface observational experiments, develop appropriate parameterization methods, and combine optimization algorithms to accurately identify surface parameters or parameter combinations, which can eventually improve the simulation capability of land-surface process models for soil moisture.
